# Effects of θ High Definition-Transcranial Alternating Current Stimulation in the Anterior Cingulate Cortex on the Dominance of Attention Focus in Standing Postural Control

**DOI:** 10.3390/bs13060477

**Published:** 2023-06-06

**Authors:** Shun Sawai, Shin Murata, Shoya Fujikawa, Ryosuke Yamamoto, Hideki Nakano

**Affiliations:** 1 Graduate School of Health Sciences, Kyoto Tachibana University, Kyoto 607-8175, Japan; sawai.neuroreha@gmail.com (S.S.); murata-s@tachibana-u.ac.jp (S.M.); fujikawa.pt@gmail.com (S.F.); shanbenliangjie201@gmail.com (R.Y.); 2 Department of Rehabilitation, Kyoto Kuno Hospital, Kyoto 607-0981, Japan; 3 Department of Physical Therapy, Faculty of Health Sciences, Kyoto Tachibana University, Kyoto 607-8175, Japan; 4 Department of Rehabilitation, Tesseikai Neurosurgical Hospital, Osaka 575-8511, Japan

**Keywords:** attention focus, dominance, standing postural control, HD-tACS, anterior cingulate cortex

## Abstract

Attention focus affects performance in postural control while standing, and it is divided into internal focus (IF) and external focus (EF). Each individual has a predominant attention focus, and research has revealed that the dominance of attention focus may be an acquired trait. However, the impact of non-invasive brain stimulation on attention-focus dominance remains unexplored in the current literature. Here, we examined the effect of high-definition transcranial alternating current stimulation (HD-tACS) on θ waves in the anterior cingulate cortex (ACC) on standing postural control tasks in an EF condition for IF- and EF-dominant groups. The effect of θ HD-tACS on the ACC differed between IF- and EF-dominant groups, and θ HD-tACS in the IF-dominant group decreased the performance of standing postural control under the EF condition. The forced activation of the ACC with θ HD-tACS may have conversely reduced the activity of brain regions normally activated by the IF-dominant group. Additionally, the activation of ACC prioritized visual information processing and suppressed the superficial sensory processing that is normally potentially prioritized by the IF-dominant group. These results highlight the importance of changing the type of rehabilitation and sports training tasks to account for the individual’s dominance of attention focus.

## 1. Introduction

When performing an action, attention focus plays a crucial role in directing the attention of an individual [1]. Attention focus can be categorized into two types, namely internal focus (IF), directing attention toward the inside of the body, and external focus (EF), directing attention towards the outside of the body. Previous studies have reported that EF conditions improve performance and have a greater motor learning effect when compared to IF conditions for the same task [2,3]. The effectiveness of EF has been examined in a variety of tasks that require accuracy, such as throwing darts [4] and putting in golf [5]. Furthermore, the effectiveness of EF has been demonstrated in standing postural control, using a stabilometer to measure the sway in center-of-gravity, resulting in a smaller sway with EF compared to that with IF [6]. Thus, the many positive effects of EF compared to IF have been reported.

Recent studies have verified the dominance of attention focus and identified two groups, namely the IF-dominant and EF-dominant groups, with positive effects on performance and motor learning when IF- or EF-dominant individuals utilize their dominant method of focus. The factors influencing the dominance of attention focus have also been examined. Studies using an upper extremity tracking task have reported that the characteristics of motor imagery ability [7], primary somatosensory cortex responses to visual and somatosensory information [8], and brain activity [9] influence the dominance of attention focus. A study comparing electroencephalogram (EEG) activity during standing postural control revealed that the IF-dominant group activated the theta waves in the inferior parietal lobule and the EF-dominant group activated theta waves in the anterior cingulate cortex (ACC) [10]. These findings indicate that each individual has a predominant attention focus, and the neural mechanisms of this focus have been studied. Additionally, past sports experience has been found to be associated with dominance in attention-focus type, with IF-dominant individuals having experienced sports in their childhood that did not involve tools, such as karate and judo, and EF-dominant individuals having experienced sports that used tools, such as soccer and tennis [7]. This suggests that the dominance of attention focus may be an acquired trait. However, no studies have investigated approaches to change the dominance of attention focus.

Non-invasive brain stimulation methods have gained attention as they can directly modulate brain activity to improve performance. Among them, transcranial alternating current stimulation (tACS) has been reported to locally modulate brain activity in a specific frequency band [11]. In addition, tACS has been reported to temporarily change cognitive function [12] and physical performance [13]. Researchers also found tACS in the theta frequency band to the midfrontal area increased immersion in virtual reality [14] and improved post-error behavioral coordination [15,16]. It has also been shown to improve working memory [17,18], memory capacity [19], and fluid intelligence [20]. More recently, high-definition tACS (HD-tACS) has been used to improve the effect of tACS. While tACS uses rectangular electrodes from 20–50 cm^2^ in area, HD-tACS uses electrodes as small as 3.14 cm^2^ to induce a more localized modulation of brain activity [21]. Therefore, HD-tACS has the potential to modulate brain activity with more precision, allowing researchers to draw more accurate data.

Past research suggests that local brain stimulation using HD-tACS could modulate the activity of brain regions related to attention-focus dominance and potentially alter this phenomenon. Additionally, modifying attention-focus dominance may enable the induction of the optimal attention-focus dominance in accordance with the specific sports category or rehabilitation task, amplifying the training effect under attention-focus conditions. However, thus far, the influence of non-invasive brain stimulation on attention-focus dominance has not been well examined in the literature. Therefore, the purpose of this study was to examine the effects of θ HD-tACS to ACC on standing postural control in IF-dominant and EF-dominant groups under EF conditions. In this study, we hypothesized that the effects of θ HD-tACS on the IF-dominant and EF-dominant groups would differ and that θ HD-tACS applied to the IF-dominant group would improve their ability to control standing posture under EF conditions. We predicted that the θ HD-tACS would alter attention-focus dominance by inducing EEG patterns similar to those of the EF-dominant group. It is anticipated that this study will contribute to the development of a novel intervention method to modify attention-focus dominance and effectively enhance the ability to control standing posture.

## 2. Materials and Methods

### 2.1. Participants

Fourteen healthy young men (age: 21.71 ± 0.47 years, height: 171.86 ± 5.63 cm, body weight: 67.14 ± 10.22 kg) with normal or corrected-to-normal vision were recruited for this study. Evidence suggests that age and gender can influence attention and brain function [22]. For the purpose of standardizing participant characteristics in this study, we exclusively recruited healthy young males. Participants with a history of motor or cognitive dysfunction were excluded. No participants fell into any other exclusionary criteria that should be considered when providing HD-tACS [23]. All participants provided informed consent, and this study was conducted in accordance with the Declaration of Helsinki. This study was approved by the local institutional ethics committee of Kyoto Tachibana University (approval no. 22-02).

### 2.2. Sample Size Calculation

The sample size was determined using G*Power software (Version: 3.1.9.6) [24]. With an effect size of 0.30, α = 0.05, and power (1 − β) = 0.80 at a confidence level of 95%, the sample size was determined to be 12.

### 2.3. Study Protocol

**Figure 1:**
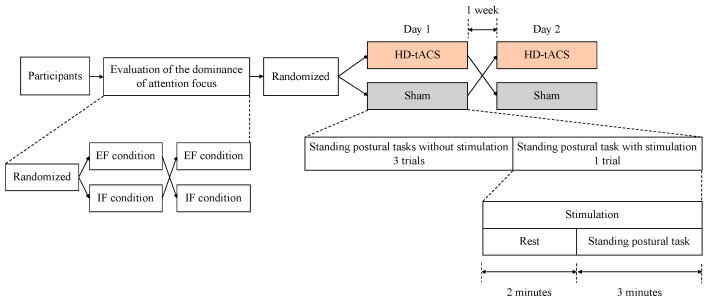
Study Protocol. This study employed a randomized, sham-controlled crossover design. Initially, all participants performed standing postural control tasks in the IF and EF conditions in random order to evaluate attention-focus dominance. Subsequently, they performed the standing postural control task in the HD-tACS and sham conditions, in random order, with a 1-week interval. In each condition, the task was first performed three times without stimulation, followed by the task in the stimulated condition. Stimulation was provided for 2 min in the resting position and for 3 min during the task. IF: internal focus; EF: external focus; HD-tACS: high-definition transcranial alternating current stimulation.

This study followed a randomized, sham-controlled, single-blinded, crossover design [25]. To evaluate the dominance of attention focus, all participants first performed the standing postural control task in the IF and EF conditions in random order. They then performed the standing postural control task in either HD-tACS or sham conditions in random order with a 1-week interval as the crossover period. The 1-week washout period was deemed sufficient, considering a prior study establishing the adequacy of a shorter, 2-day washout period [26]. In each condition, the participants performed the standing posture control task in the stimulated condition after three sessions of the standing posture control task without brain stimulation as practice for the task. During the standing posture control task that provided brain stimulation, the participants were first stimulated in a resting position for 2 min and then performed the standing posture control task in a stimulated state for 3 min (Figure 1). We intentionally omitted periods of ramping up stimulus intensity from the task. It should also be noted that this pre-stimulation protocol, designed to enhance the efficacy of tACS, has been employed in prior studies [27]. In the present study, all tasks were performed in the EF condition except when determining attention-focus dominance.

### 2.4. Standing Postural Task

**Figure 2:**
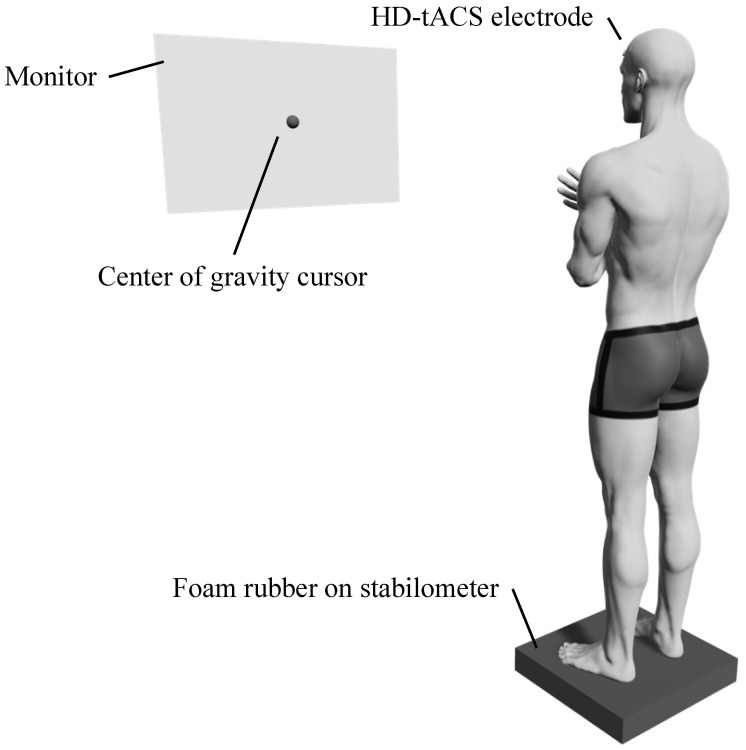
Experimental Setup. Participants stood barefoot on a stabilometer with foam rubber on top, crossing their arms in front of their chest. A monitor was positioned at eye level in front of the participant, displaying the real-time center of gravity measured with the stabilometer. During the task in the stimulus condition, participants wore HD-tACS electrodes on their heads, which provided the respective stimuli. HD-tACS: high-definition transcranial alternating current stimulation.

**Figure 3:**
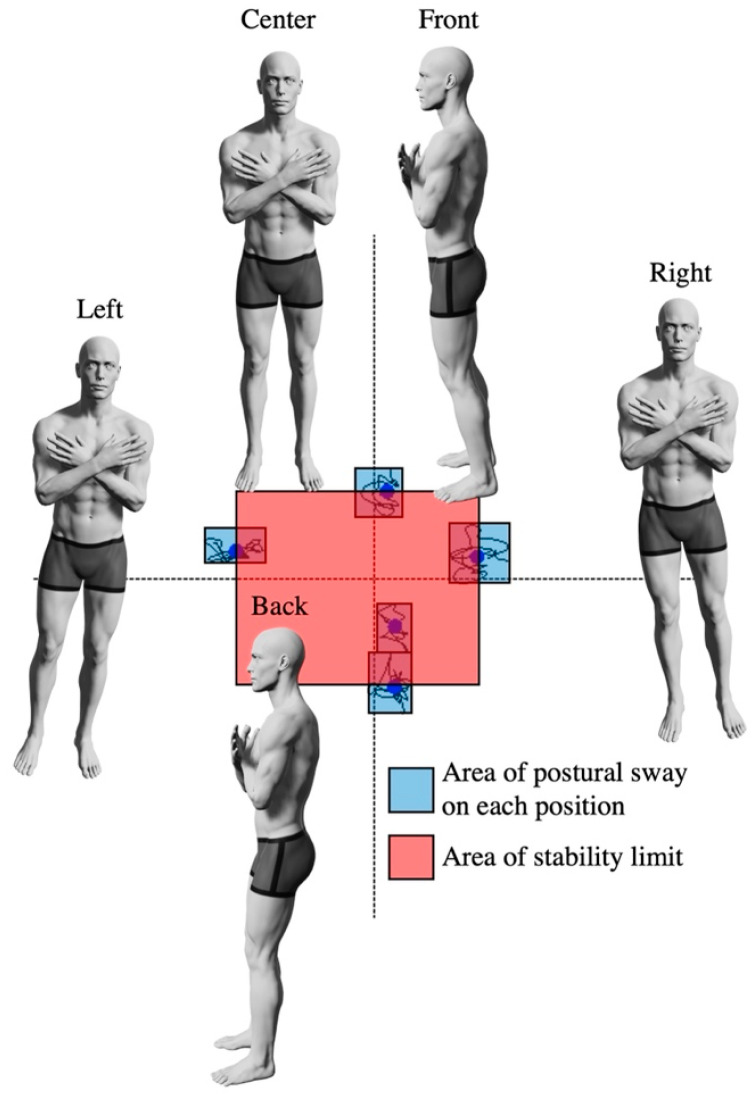
Index of Postural Stability. Participants initially measured their center of gravity sway while standing stationary at the center for 10 s. Subsequently, they maintained their posture for 10 s with the center of gravity shifted to its maximum in the front, back, left, and right directions, and the center of gravity sway was assessed. The blue area represents the postural sway area, while the red area indicates the stability limit area. The postural sway area is the average for each rectangular region. The stability limit area is calculated based on the distance between the centers of each rectangle.

The index of postural stability (IPS) [10,28] was selected as the standing posture control task in this study. Participants stood barefoot on a stabilometer (T.K.K.5810; Takei Kiki Kogyo Co., Ltd., Niigata, Japan) with foam rubber (ANIMA Co., Ltd., Tokyo, Japan) on top, with their arms crossed in front of their chest. A monitor was placed in front of the participant at eye level, and the position of the center of gravity measured with the stabilometer was displayed in real time (Figure 2). The center of gravity sway was measured for 10 s in the central static standing position; then positions were adjusted to shift the center of gravity toward the front, back, right, and to the left, and the center of gravity sway was also measured for 10 s in each of these positions (Figure 3). The order of measurement was the same for all participants.

The IPS requires a challenging standing postural control task. This index of measurement was chosen due to its ability to measure postural sway more accurately, with no upper limit on the achievable score, especially in individuals aged <30 years [10,28]. This would help negate any other confounding factors that were not accounted for by the inclusion or exclusion criteria. At this time, participants were instructed to “pay attention to the center of gravity point displayed on the monitor, move the point as far up (down, right or left based on the posture being tested) as possible, and hold it for 10 s” to focus their attention on the external body [10]. After the task, participants self-evaluated their ability to pay attention to the verbal instructions based on the Numerical rating scale (0–100). Participants who scored less than 60 were excluded from this study because they could not pay attention to the verbal instructions [10,29].

### 2.5. Dominance of Attention Focus

To evaluate the dominance of attention focus, IPS was conducted in the IF condition in addition to the EF condition described above. In the IF condition, participants were instructed to “focus their attention on their feet, place their weight on the front (back, right or left based on the posture being tested) of their feet and hold it for 10 s”. The participants were asked to focus their attention on the inside of their bodies [10]. Then, IPS scores were computed and compared in the IF and EF conditions. Participants with higher IPS scores in the IF condition were defined as the IF-dominant group, whereas participants with higher IPS scores in the EF condition were defined as the EF-dominant group [7,10].

### 2.6. HD-tACS

**Figure 4:**
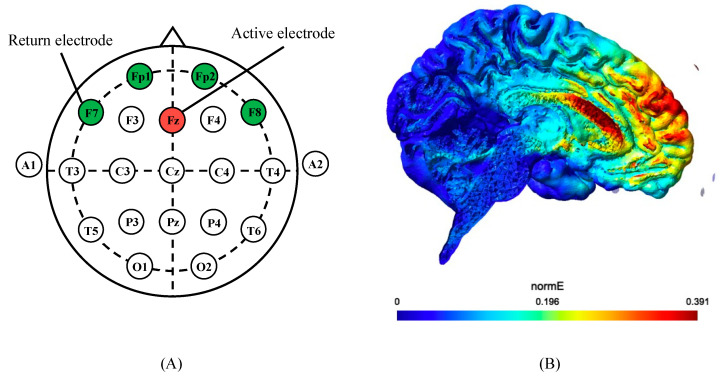
Electrode setup. (**A**) The active electrode was attached to Fz and the return electrode to F7, Fp1, Fp2, and F8 according to the international 10–20 method. (**B**) Stimulus modeling using Sim NIBS confirmed that modulation of the electric field occurs at ACC.

HD-tACS was delivered using a Nurostym tES device, courtesy of Neuro Device S.A., Warszawa, Poland. This setup included Sintered Ag/AgCl electrodes with a 1 cm radius and an electrolyte gel provided by Brain Produst GmbH, Gilching, Germany, which served to enhance impedance reduction. The electrodes were placed on the scalp according to the international 10–20 method (Figure 4A) [30]. The electrodes were arranged in a 4 × 1 HD-tACS configuration consisting of one active electrode and four return electrodes. The placement of the electrodes was derived after referencing multiple studies that used and compared high-definition transcranial direct-current stimulation (HD-tDCS) with HD-tACS with the same electrode placement, targeting the same brain areas as in this study, was used successfully [31]. This placement was therefore adopted. The active electrode was placed at Fz, and the return electrodes at Fp1, Fp2, F7, and F8, following the standard [30]. This modulated the activity of the ACC, which was activated in the EF-dominant group [10].

Stimulus intensity was set to 2.0 mA, and the stimulus frequency was set to 6.5 Hz in the theta wave band. This stimulus was used on the EF-dominant group in a previous study [10] and adapted to this study as well. The stimulation duration was 5 min, including a 10 s ramp-up/ramp-down period. In all cases, we utilized online stimulations due to their established superiority in effectiveness over offline stimulations [32].

In contrast, the sham group received a 10 s ramp-up and ramp-down stimulus at the beginning and end with no stimulation between the ramp-up and ramp-down periods. Impedance was maintained below 15 kΩ during stimulation [33].

The “Simulation of Non-Invasive Brain Stimulation” (SimNIBS, http://simnibs.org/, accessed on 1 April 2023, version: 3.2.6) to model electrical stimulation was used, which confirmed the modulation of the electric field in the ACC (Figure 4B). For the electrical simulation, we utilized the standardized ‘Ernie’ head model provided within the SimNIBS software suite.

### 2.7. Measures

The area of postural sway and area of stability limit were calculated from the measured center-of-gravity sway data. The area of postural sway is the average of the rectangular areas created by the postural sway from each of the five different positions of the center of gravity. The area of stability limit was calculated as “front and rear center of gravity movement distance between anterior and posterior positions × the distance between right and left positions” (Figure 3). The IPS was then calculated using the following Equation (1) [28].

(1) $$IPS=\log\frac{\left(area\;of\;stability\;limit\;+\;area\;of\;postural\;sway\right)}{area\;of\;postural\;sway}$$

In addition, to account for the diurnal variation in performance of the postural control task, the IPS change rate was calculated using the following Equation (2), using IPS in the third condition without stimulation:

(2) $$IPS\;change\;rate\;=\;\frac{IPS\;in\;the\;stimulation\;condition}{IPS\;in\;the\;third\;condition\;without\;stimulation}$$

### 2.8. Statistical Analysis

First, the normality of the data was confirmed using the Shapiro–Wilk test. Then, a paired *t*-test was performed on the IPS under the stimulation condition, the IPS under the third condition without stimulation, and the IPS change rate for all participants. The IPS change rates were compared using two-factor repeated-measures analysis of variance (ANOVA) using the following two factors: group (IF- and EF-dominant groups) and condition (HD-tACS and sham conditions). Post hoc tests were conducted using the Bonferroni method. All statistical analyses were performed using SPSS ver. 28.0 (IBM Corp., Armonk, NY, USA), and the significance level was set at *p* < 0.05.

## 3. Results

**Figure 5:**
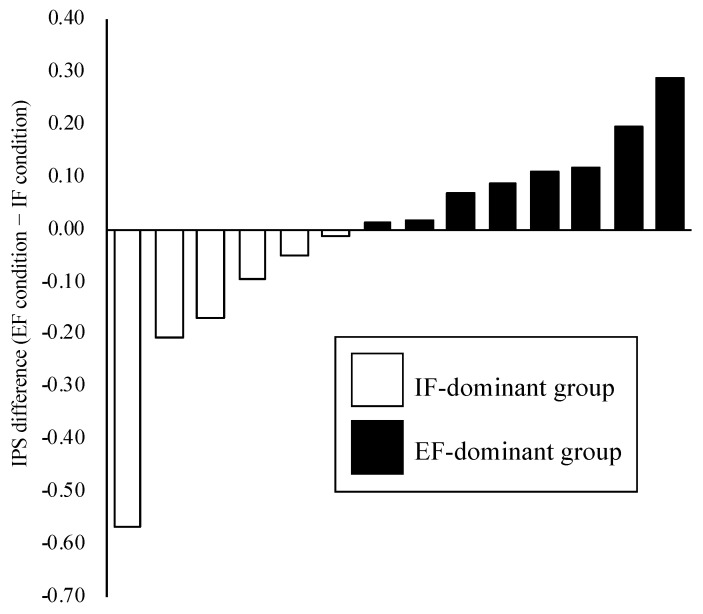
Attention-Focus Dominance. To assess attention-focus dominance, the IPS was measured in both IF and EF conditions. Participants were then divided into an IF-dominant group (*n* = 6), which exhibited better performance in the IF condition, and an EF-dominant group (*n* = 8), which demonstrated superior performance in the EF condition. IPS: index of postural stability; IF: internal focus; EF: external focus.

**Figure 6:**
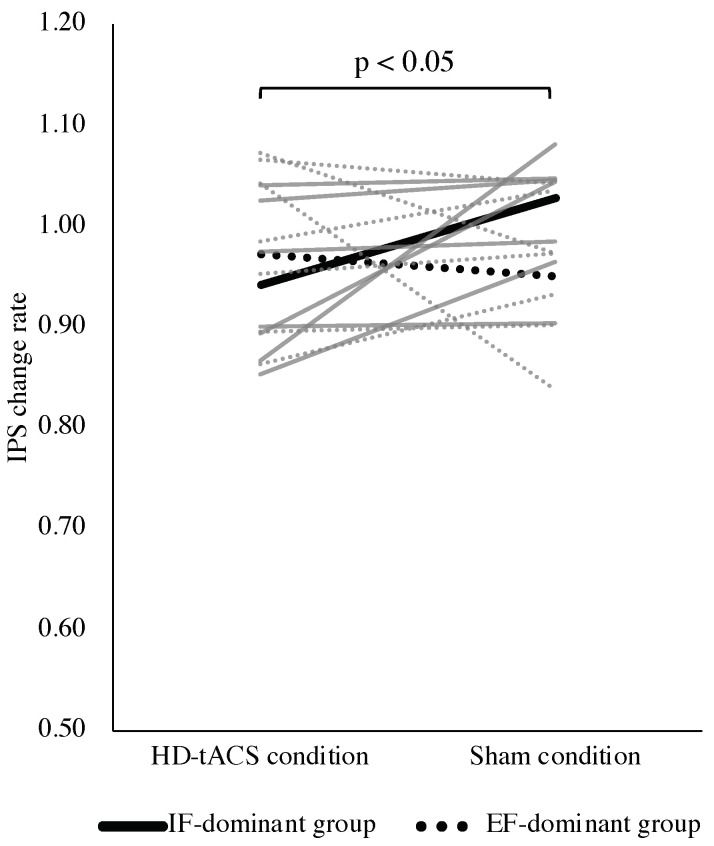
Comparison of IPS change rate between groups. The solid line represents the IF-dominant group, while the dotted line represents the EF-dominant group. Gray lines display individual participant data, and black lines represent the mean value for each group. A two-factor repeated-measures analysis of variance indicated a significant interaction between group and condition (*p* < 0.05). Post hoc tests showed that the IPS change rate for the IF-dominant group was significantly lower in the HD-tACS condition compared to the sham condition (*p* < 0.05). In contrast, no significant difference was found in the IPS change rate for the EF-dominant group between conditions (*p* > 0.05). The IPS change rate in the sham condition was significantly higher for the IF-dominant group than the EF-dominant group. IPS: index of postural stability; HD-tACS: high-definition transcranial alternating current stimulation; IF: internal focus; EF: external focus.

The participants were initially classified into IF-dominant (*n* = 6) and EF-dominant (*n* = 8) groups based on the assessment of attention-focus dominance (Figure 5). The paired *t*-tests showed no significant difference in IPS under the stimulation condition and IPS change rate between the HD-tACS and sham conditions (Table 1). However, the two-factor repeated-measures ANOVA demonstrated a significant interaction between group and condition in the IPS change rate (F = 5.04, *p* = 0.04). Subsequent post hoc analysis revealed that the IPS change rate in the HD-tACS condition was significantly lower in the IF-dominant group than in the sham condition (F = 5.57, *p* = 0.04) (Figure 6). Conversely, no significant differences were observed in the IPS change rate between the conditions in the EF-dominant group (F = 0.50, *p* = 0.49). Notably, the IPS change rate in the sham condition was significantly higher in the IF-dominant group than in the EF-dominant group (F = 5.64, *p* = 0.04). There no adverse events due to HD-tACS were observed.

## 4. Discussion

The aim of this study was to investigate the effects of θ HD-tACS on standing postural control in the ACC under the EF condition in IF-dominant and EF-dominant groups. A paired *t*-test showed that the IPS change rate did not significantly differ between the HD-tACS and sham conditions. However, a two-factor repeated-measures ANOVA showed an interaction between group and condition in the IPS change rate. Additionally, the post hoc tests for the IF-dominant group revealed that the IPS change rate in the HD-tACS condition was significantly lower than that in the sham condition, while the IPS change rate in the EF-dominant group did not differ significantly between conditions. It has been documented that in the EF-dominant group, the θ wave band frequency in the ACC is activated and is linked with the predominance of attention focus [10]. Consequently, our findings suggest that the impact of θ HD-tACS on the ACC varies between the IF-dominant and EF-dominant groups. Moreover, these results underscore the significance of implementing interventions under conditions that are optimal, guided by the predominance of attention focus, as opposed to immediate modulation of brain activity with electrical stimulation.

### 4.1. Effect of θ HD-tACS on Standing Postural Control under EF Conditions

The IPS in the third condition without stimulation, IPS in the stimulation condition, and IPS change rate did not significantly differ between the HD-tACS and sham conditions, indicating that θ HD-tACS does not improve the performance of standing postural control in the EF condition. This result may be attributed to the fact that the effect of θ HD-tACS may not be the same for all participants. Previous studies have shown that each individual has a dominance of attention focus that improves performance, and factors related to the dominance of attention focus have been examined [7,8,9,10]. Therefore, the effect of θ HD-tACS may have varied depending on individual differences in this attentional function. Additionally, previous studies have reported that the effect of tACS on cognitive function in healthy young adults is present but is small [19]. Therefore, it is possible that the θ HD-tACS did not cause a significant change in standing postural control performance in this study and that the effect of the θ HD-tACS differed depending on the characteristics of the participants and other factors.

### 4.2. Effect of θ HD-tACS on Standing Postural Control under EF Conditions in IF- and EF-Dominant Groups

The results presented above allowed the categorization of participants into IF- and EF-dominant groups and the comparison of the effects of θ HD-tACS. A significant interaction between the group (IF- and EF-dominant groups) and condition (HD-tACS and sham conditions) was observed for the IPS change rate through the two-factor repeated-measures ANOVA. In the IF-dominant group, the IPS rate change in the HD-tACS condition was significantly lower than that in the sham condition. However, no significant difference in the IPS change rate was observed between conditions for the EF-dominant group.

These findings suggest that the effect of θ HD-tACS on the ACC differs between IF- and EF-dominant groups and that θ HD-tACS in the IF-dominant group decreases the performance of standing postural control under the EF condition. Previous research showed that during tasks in the EF condition, ACC activation was higher in the EF-dominant group compared to the IF-dominant group [10]. Additionally, ACC involvement in error detection based on visual information [34] suggests that it may represent brain functional characteristics of the EF-dominant group. Some researchers have proposed that the dominance of attention focus could be an acquired trait [7]. We hypothesized that an immediate change in ACC activity to a state similar to that of the EF-dominant group would result in an immediate improvement in standing postural control performance under the EF condition. Contrary to our hypothesis, θ HD-tACS decreased the performance of standing postural control under EF conditions for the IF-dominant group in this study.

This outcome may be attributed to the HD-tACS forcing the IF-dominant group to use a brain region not typically engaged for standing postural control, potentially reducing their performance. Previous studies have demonstrated that tACS to relevant regions in cognitive functions, such as working memory, improves their function [35,36]. Conversely, tACS to the parietal lobe, which is unrelated to working memory, has been shown to decrease working memory capacity [37]. These results suggest that tACS to nonrelevant regions may impair performance. In the present study, θ activity in the ACC might have been highly active during standing postural control in the EF condition for the EF-dominant group but not related to standing postural control for the IF-dominant group. Thus, we speculate that θ HD-tACS forced the ACC, which was not involved in standing postural control of the IF-dominant group, to become more active, thereby decreasing the ability of the IF-dominant group to control standing posture under the EF condition.

It has also been reported that the IF-dominant group suppresses other stimuli, such as visual information, and implicitly implements a superficial sensory-dominant postural control because the processing of the superficial senses takes priority [8]. This feature is explained with a sensory gating system in which the second stimulus-response is significantly reduced in response to the first stimulus-response [38,39]. In this study, it is possible that the θ HD-tACS activated the ACC, which prioritized the processing of visual stimuli in the same pattern as the EF-dominant group. It is speculated that this reduced the ability of the IF-dominant group to control standing posture by decreasing the superficial sensory response, which is normally processed preferentially by the IF-dominant group.

On the contrary, the performance of standing postural control in the EF-dominant group remained unchanged by HD-tACS. For working memory, tACS has been reported to have significant effects on participants with reduced working memory and brain activity in related areas, commonly found in the elderly and patients with neurological diseases [36]. These results suggest that tACS may have a small effect on participants with high performance and sufficiently activated EEG. In this study, the EF-dominant group had more consistent standing postural control under EF conditions than the IF-dominant group, suggesting that the ACC may have already been activated. Therefore, it is speculated that HD-tACS on the ACC did not change the performance of standing postural control under EF conditions. These results indicate the need to tailor tasks according to each individual’s attention-focus dominance rather than using transcranial brain stimulation or other methods to change the dominance of attention focus according to the type of task in rehabilitation or sports training.

### 4.3. Potential Standing Postural Control Ability of IF-Dominant and EF-Dominant Groups

The IPS change rate in the sham condition was significantly greater in the IF-dominant group compared to the EF-dominant group. This outcome suggests that the IF-dominant group demonstrated a higher potential capability for standing postural control than the EF-dominant group. Prior research has indicated that focusing on internal bodily sensations is cognitively demanding, which implies that the IF-dominant group possesses advanced cognitive function [9]. Additionally, it has been noted that attention-focus dominance is determined not by reaction speed to visual stimuli but by response speed to superficial sensory stimuli, suggesting a distinct attentional function in the IF-dominant group [8]. In a previous investigation, standing postural control performance in the EF condition was significantly better in the IF-dominant group than in the EF-dominant group, although no significant difference was observed between IF- and EF-dominant groups in the EF condition [10]. The outcomes of the prior study imply that the IF-dominant group may exhibit superior potential standing postural control under attention-focus conditions compared to the EF-dominant group. Given these observations, it is possible that both potential standing postural control and attentional control abilities were greater in the IF-dominant group than in the EF-dominant group in the current study. As a result, it can be speculated that the IF-dominant group outperformed the EF-dominant group in the sham condition.

### 4.4. Limitations

This study has several limitations: First, EEG measurements were not conducted before and after stimulation, rendering it impossible to directly confirm whether ACC activity was modulated with θ HD-tACS. Although the electrode configuration employed in this study has been utilized in previous research and is anticipated to modulate ACC activity in current simulations, actual EEG modulation with θ HD-tACS remains unverified due to the lack of measurements. Additionally, our inability to determine individual responsiveness to HD-tACS is compounded by the absence of EEG measurements. As previous research indicates, responses to noninvasive brain stimulation methods exhibit substantial variability between individuals [40]. Consequently, subsequent studies should aim to confirm the adaptation to HD-tACS by conducting prestimulation and poststimulation EEG measurements. Second, θ HD-tACS might have affected regions other than the ACC. θ HD-tACS has smaller electrodes compared to those of tACS, allowing for more localized stimulation. However, current simulations predicted that stimulation effects would extend to brain regions surrounding the ACC, which may have influenced this study’s outcomes. Future research could address this limitation by using personalized stimulus montages [41]. Third, it remains unclear whether attention was accurately directed to the body’s exterior. The sole evaluation scale for attention focus is a subjective percentage rating [29], and it is necessary to develop an objective scale for evaluating attention location more precisely in future research. Fourth, our investigation was limited to the immediate effects and overlooked the long-term consequences. For a more comprehensive understanding, future studies should illuminate not only the long-term effects but also their persistence over time.

## 5. Conclusions

This study aimed to examine the effect of θ HD-tACS on ACC and standing postural control under EF conditions in IF-dominant and EF-dominant groups. The results demonstrated that θ HD-tACS to ACC decreased standing postural control performance in the EF condition for the IF-dominant group. One possible explanation for this outcome is that θ HD-tACS forced the IF-dominant group to activate brain regions not typically used by them, resulting in reduced activity in brain regions normally employed by the IF-dominant group and potentially decreased performance. Another possibility is that θ HD-tACS activated the ACC, prioritizing visual information processing and reducing superficial sensory responses, which are normally preferentially processed by the IF-dominant group, leading to diminished performance. These findings suggest that requiring attentional strategies that differ from an individual’s attention-focus dominance can impair performance. Therefore, it highlights the importance of adapting the type of rehabilitation and sports training tasks to accommodate each individual’s attention-focus dominance. Future studies should examine the effects of attention-focus dominance on motor learning and further encourage its use in clinical treatment. Furthermore, effective rehabilitation and sports training interventions should be developed to identify individually optimized intervention methods based on the attention-focus dominance.

## Figures and Tables

**Table 1:** Results of IPS between each condition.

	HD-tACS Condition	Sham Condition	*p*-Value
IPS in the third condition without stimulation	1.83 ± 0.22	1.85 ± 0.19	0.77
IPS in the stimulation condition	1.75 ± 0.21	1.81 ± 0.21	0.17
IPS change rate	0.96 ± 0.08	0.98 ± 0.07	0.39

IPS: Index of postural stability; HD-tACS: High-definition transcranial alternating stimulation.

## Data Availability

The data that support the findings of this study are available on request from the corresponding author. The data are not publicly available because they contain information that can compromise the privacy of research participants.

## References

[1] Wulf G., Höß M., Prinz W. (1998). Instructions for motor learning: Differential effects of internal versus external focus of attention. J. Mot. Behav..

[2] Wulf G., Shea C., Lewthwaite R. (2010). Motor skill learning and performance: A review of influential factors. Med. Educ..

[3] Park S.H., Yi C.W., Shin J.Y., Ryu Y.U. (2015). Effects of external focus of attention on balance: A short review. J. Phys. Ther. Sci..

[4] Hitchcock D.R., Sherwood D.E. (2018). Effects of Changing the Focus of Attention on Accuracy, Acceleration, and Electromyography in Dart Throwing. Int. J. Exerc. Sci..

[5] Wulf G., Lauterbach B., Toole T. (1999). The learning advantages of an external focus of attention in golf. Res. Q. Exerc. Sport.

[6] Chiviacowsky S., Wulf G., Wally R. (2010). An external focus of attention enhances balance learning in older adults. Gait. Posture.

[7] Sakurada T., Hirai M., Watanabe E. (2016). Optimization of a motor learning attention-directing strategy based on an individual’s motor imagery ability. Exp. Brain Res..

[8] Sakurada T., Yoshida M., Nagai K. (2022). Individual Optimal Attentional Strategy in Motor Learning Tasks Characterized by Steady-State Somatosensory and Visual Evoked Potentials. Front. Hum. Neurosci..

[9] Sakurada T., Hirai M., Watanabe E. (2019). Individual optimal attentional strategy during implicit motor learning boosts frontoparietal neural processing efficiency: A functional near-infrared spectroscopy study. Brain Behav..

[10] Sawai S., Fujikawa S., Murata S., Abiko T., Nakano H. (2022). Dominance of Attention Focus and Its Electroencephalogram Activity in Standing Postural Control in Healthy Young Adults. Brain Sci..

[11] Elyamany O., Leicht G., Herrmann C.S., Mulert C. (2021). Transcranial alternating current stimulation (tACS): From basic mechanisms towards first applications in psychiatry. Eur. Arch. Psychiatry Clin. Neurosci..

[12] Klink K., Paßmann S., Kasten F.H., Peter J. (2020). The Modulation of Cognitive Performance with Transcranial Alternating Current Stimulation: A Systematic Review of Frequency-Specific Effects. Brain Sci..

[13] Sale M.V., Kuzovina A. (2022). Motor training is improved by concurrent application of slow oscillating transcranial alternating current stimulation to motor cortex. BMC Neurosci..

[14] Boukarras S., Özkan D.G., Era V., Moreau Q., Tieri G., Candidi M. (2022). Midfrontal Theta Transcranial Alternating Current Stimulation Facilitates Motor Coordination in Dyadic Human-Avatar Interactions. J. Cogn. Neurosci..

[15] Fusco G., Scandola M., Feurra M., Pavone E.F., Rossi S., Aglioti S.M. (2018). Midfrontal theta transcranial alternating current stimulation modulates behavioural adjustment after error execution. Eur. J. Neurosci..

[16] Fusco G., Cristiano A., Perazzini A., Aglioti S.M. (2022). Neuromodulating the performance monitoring network during conflict and error processing in healthy populations: Insights from transcranial electric stimulation studies. Front. Integr. Neurosci..

[17] Hu Z., Samuel I.B.H., Meyyappan S., Bo K., Rana C., Ding M. (2022). Aftereffects of frontoparietal theta tACS on verbal working memory: Behavioral and neurophysiological analysis. IBRO Neurosci. Rep..

[18] Kvašňák E., Magyarová E., Domankuš M., Tesař M., Kymplová J., Fetissov V., Abubaker M., Al Qasem W. (2022). 10 Minutes Frontal 40 Hz tACS-Effects on Working Memory Tested by Luck-Vogel Task. Behav. Sci..

[19] Booth S.J., Taylor J.R., Brown L.J.E., Pobric G. (2022). The effects of transcranial alternating current stimulation on memory performance in healthy adults: A systematic review. Cortex.

[20] Pahor A., Jaušovec N. (2014). The effects of theta transcranial alternating current stimulation (tACS) on fluid intelligence. Int. J. Psychophysiol..

[21] Ghafoor U., Yang D., Hong K.S. (2022). Neuromodulatory Effects of HD-tACS/tDCS on the Prefrontal Cortex: A Resting-State fNIRS-EEG Study. IEEE J. Biomed. Health Inform..

[22] Rubia K., Hyde Z., Halari R., Giampietro V., Smith A. (2010). Effects of age and sex on developmental neural networks of visual-spatial attention allocation. Neuroimage.

[23] Antal A., Alekseichuk I., Bikson M., Brockmöller J., Brunoni A.R., Chen R., Cohen L.G., Dowthwaite G., Ellrich J., Flöel A. (2017). Low intensity transcranial electric stimulation: Safety, ethical, legal regulatory and application guidelines. Clin. Neurophysiol..

[24] Faul F., Erdfelder E., Lang A.G., Buchner A. (2007). G*Power 3: A flexible statistical power analysis program for the social, behavioral, and biomedical sciences. Behav. Res. Methods.

[25] Benussi A., Cantoni V., Cotelli M.S., Cotelli M., Brattini C., Datta A., Thomas C., Santarnecchi E., Pascual-Leone A., Borroni B. (2021). Exposure to gamma tACS in Alzheimer’s disease: A randomized, double-blind, sham-controlled, crossover, pilot study. Brain Stimul..

[26] Mansouri F., Shanbour A., Mazza F., Fettes P., Zariffa J., Downar J. (2019). Effect of Theta Transcranial Alternating Current Stimulation and Phase-Locked Transcranial Pulsed Current Stimulation on Learning and Cognitive Control. Front. Neurosci..

[27] Moliadze V., Stenner T., Matern S., Siniatchkin M., Nees F., Hartwigsen G. (2021). Online Effects of Beta-tACS Over the Left Prefrontal Cortex on Phonological Decisions. Neuroscience.

[28] Suzuki Y., Yatoh S., Suzuki H., Tanabe Y., Shimizu Y., Hada Y., Shimano H. (2018). Age-dependent changes in dynamic standing-balance ability evaluated quantitatively using a stabilometer. J. Phys. Ther. Sci..

[29] Richer N., Polskaia N., Lajoie Y. (2017). Continuous Cognitive Task Promotes Greater Postural Stability than an Internal or External Focus of Attention in Older Adults. Exp. Aging Res..

[30] To W.T., Eroh J., Hart J., Vanneste S. (2018). Exploring the effects of anodal and cathodal high definition transcranial direct current stimulation targeting the dorsal anterior cingulate cortex. Sci. Rep..

[31] Lang S., Gan L.S., Alrazi T., Monchi O. (2019). Theta band high definition transcranial alternating current stimulation, but not transcranial direct current stimulation, improves associative memory performance. Sci. Rep..

[32] Pozdniakov I., Vorobiova A.N., Galli G., Rossi S., Feurra M. (2021). Online and offline effects of transcranial alternating current stimulation of the primary motor cortex. Sci. Rep..

[33] Lehr A., Henneberg N., Nigam T., Paulus W., Antal A. (2019). Modulation of Conflict Processing by Theta-Range tACS over the Dorsolateral Prefrontal Cortex. Neural Plast..

[34] Slobounov S.M., Fukada K., Simon R., Rearick M., Ray W. (2000). Neurophysiological and behavioral indices of time pressure effects on visuomotor task performance. Brain Res. Cogn. Brain Res..

[35] Abubaker M., Al Qasem W., Kvašňák E. (2021). Working Memory and Cross-Frequency Coupling of Neuronal Oscillations. Front. Psychol..

[36] Al Qasem W., Abubaker M., Kvašňák E. (2022). Working Memory and Transcranial-Alternating Current Stimulation-State of the Art: Findings, Missing, and Challenges. Front. Psychol..

[37] Möller A., Nemmi F., Karlsson K., Klingberg T. (2017). Transcranial Electric Stimulation Can Impair Gains during Working Memory Training and Affects the Resting State Connectivity. Front. Hum. Neurosci..

[38] Cromwell H.C., Mears R.P., Wan L., Boutros N.N. (2008). Sensory gating: A translational effort from basic to clinical science. Clin. EEG Neurosci..

[39] Wiesman A.I., Heinrichs-Graham E., Coolidge N.M., Gehringer J.E., Kurz M.J., Wilson T.W. (2017). Oscillatory dynamics and functional connectivity during gating of primary somatosensory responses. J. Physiol..

[40] López-Alonso V., Cheeran B., Río-Rodríguez D., Fernández-Del-Olmo M. (2014). Inter-individual variability in response to non-invasive brain stimulation paradigms. Brain Stimul..

[41] Vohryzek J., Cabral J., Castaldo F., Sanz-Perl Y., Lord L.D., Fernandes H.M., Litvak V., Kringelbach M.L., Deco G. (2022). Dynamic sensitivity analysis: Defining personalised strategies to drive brain state transitions via whole brain modelling. Comput. Struct. Biotechnol. J..

